# Implementing a national diabetes prevention programme in England: lessons learned

**DOI:** 10.1186/s12913-019-4809-3

**Published:** 2019-12-23

**Authors:** Jonathan Stokes, Judith Gellatly, Peter Bower, Rachel Meacock, Sarah Cotterill, Matt Sutton, Paul Wilson

**Affiliations:** 1 Centre for Primary Care and Health Services Research, University of Manchester, Manchester, UK; 2 Division of Nursing, University of Manchester, Manchester, UK; 3 Centre for Biostatistics, University of Manchester, Manchester, UK; 4 Alliance Manchester Business School, University of Manchester, Manchester, UK

**Keywords:** Diabetes, Prevention, Implementation, Health systems

## Abstract

**Background:**

Type 2 diabetes mellitus is preventable through lifestyle intervention. Diabetes prevention programmes (DPPs) aim to deliver prevention-based behaviour change interventions to reduce incidence. Such programmes vary from usual primary care in terms of where, how, and by whom they are delivered. Implementation is therefore likely to face new commissioning, incentive and delivery challenges. We report on the implementation of a national DPP in NHS England, and identify lessons learned in addressing the implementation challenges.

**Methods:**

In 2017/18, we conducted 20 semi-structured telephone interviews covering 16 sampled case sites with the designated lead(s) responsible for local implementation of the programme. Interviews explored the process of implementation, including organisation of the programme, expectations and attitudes to the programme, funding, target populations and referral and clinical pathways. We drew on constant comparative methods to analyse the data and generate over-arching themes. We complemented our qualitative data with a survey focused on variation in the financial incentives used across sites to ensure usual primary care services recruited patients to new providers.

**Results:**

We identified five over-arching areas of learning for implementing this large-scale programme: 1) managing new providers; 2) promoting awareness of services; 3) recruiting patients; 4) incentive payments; and 5) mechanisms for sharing learning. In general, tensions appeared to be caused by a lack of clear roles/responsibilities between hierarchical actors, and lack of communication. Both local sites and the national NHS coordination team gained experience through learning by doing. Initial tensions with roles and expectations have been worked out during implementation.

**Conclusions:**

Implementing a national disease prevention programme is a major task, and one that will be increasingly faced by health systems globally as they aim to adjust to demand pressures. We provide practical learning opportunities for the wider uptake and sustainability of prevention programmes. Future implementers might wish to define clear responsibilities for each actor prior to implementation, ensure early engagement with new providers, offer mechanisms/forums for sharing learning, generate evidence and provide advice on incentive payments, and prioritise public and professional awareness of the programme.

## Background

The focus on prevention of long-term conditions is increasing globally. The recent World Health Organization’s (WHO’s) Astana Declaration reaffirmed the commitment of member states to “prioritize disease prevention and health promotion” [1]. In the UK, the National Health Service (NHS) Long Term Plan endorsed “more NHS action on prevention and health inequalities” [2].

Diabetes is one of four priority long-term conditions targeted by the WHO [3]. For an individual, developing type 2 diabetes mellitus can result in loss of vision, nerve pain, and in severe cases, limb amputation [4]. Type 2 diabetes is also associated with an increased risk of developing further cardiovascular diseases and multimorbidity [4]. In the UK diabetes is estimated to cost the NHS £9.8 billion per year in direct treatment costs, close to 10% of the total health system budget, and the UK economy a further £13.9 billion in indirect social and productivity costs [5]. However, type 2 diabetes is largely preventable, with lifestyle intervention (behaviour change to achieve a healthy diet, weight loss and exercise) more effective than drugs for reducing risk [6–9].

Diabetes Prevention Programmes (DPPs) have been developed and implemented worldwide since the 1990s [10]. Separate randomised controlled trials of DPP programmes in Finland [7] and the USA [6] both showed diabetes incidence could be reduced through behaviour change programmes aimed at weight-loss.

There is a question of when, where, how, and by whom these expanding prevention services should be planned and delivered. The Finnish DPP was primarily delivered by nutritionists, with diet and exercise advice delivered face-to-face in both individual and group sessions [11]. The USA DPP involved similar activities delivered by ‘lifestyle coaches’, the majority of whom were trained dieticians [12]. Since these early studies were conducted, DPPs implemented worldwide have varied in design and programme intensity [13–16].

In England, the Healthier You: NHS DPP is an ambitious national programme aiming to deliver an evidence-based behaviour change intervention to patients at risk of developing diabetes. The programme targets individuals with non-diabetic hyperglycaemia or ‘pre-diabetes’, who have raised blood glucose levels but do not yet fall in the diabetic range. People with pre-diabetes are identified through general practice (GP) patient registers and NHS Health Checks which are offered every 5 years for 40–74 year olds. Patients who have been diagnosed to be at risk by an HbA1c blood glucose test are referred to the DPP by their GP. It is then the patient’s choice whether they take up the service. The core DPP intervention offers group-based delivery of a course of sessions offering behaviour change content to achieve dietary change, physical activity and weight loss. The course consists of a minimum of 16 h of contact time over at least 9 months [17].

A strong GP-led primary care system exists in England, which is often seen as a core platform for prevention but involvement of professionals such as nutritionists or lifestyle coaches is unusual. Instead of adding the prevention programme into existing primary care services, the Healthier You programme chose to implement the DPP through four provider organisations, three of which are privately-run and one is from the third sector. This is a marked change to traditional primary care delivery, adding an additional layer of providers to the health system and separating diabetes prevention from other prevention programmes and from routine primary care delivery. It is therefore likely to face new commissioning, incentive and delivery opportunities and challenges.

Previous research has evaluated the pilot NHS DPP demonstrator sites, reporting on intervention fidelity and recruitment and retention challenges [18, 19]. To date, there has been no examination of the macro-level implementation challenges of scaling up the DPP for national provision. We are currently evaluating the effectiveness of the national roll-out of the NHS DPP through a mixed methods evaluation, the DIPLOMA (Diabetes Prevention – Long term Multimethod Assessment) research programme [20]. This paper draws on results from the work package evaluating the process of programme implementation.

Understanding why interventions can be successfully implemented in some settings but not others is a key issue for the wider uptake and sustainability of programmes like the NHS DPP. Here we report on our qualitative and survey findings around national DPP implementation in England, and provide lessons learned to address the challenges faced. First, we provide more detailed background on the commissioning and delivery of the DPP in England.

### Commissioning the DPP in England

#### Commissioning arrangements for prevention activity in the NHS in England

Understanding the commissioning arrangements in the NHS in England is important for comprehending the implementation challenges we identified for the DPP.

Commissioning (planning and strategic purchasing) of services has been evolving in the NHS in England since 1991 when the internal market was first introduced [21]. Just over 200 Clinical Commissioning Groups (CCGs) were established in 2013 [22]. These are statutory NHS bodies led by primary care practitioners that plan and commission health care services for their local area, and are responsible for allocating nearly two thirds of the total NHS budget in England [23]. However, public health commissioning has remained largely outside of the remit of CCGs, remaining either nationally commissioned by NHS England in conjunction with Public Health England or, as in the case in areas like sexual health, through whole system commissioning involving a mix of local and national agencies [24].

Previous research suggests these commissioning arrangements might limit large-scale change in health services, particularly integration between multiple providers, as it may limit provider engagement and a focus on implementation, favouring transactional rather than relational dimensions [23]. Different contracting arrangements for each provider across a care pathway can make co-ordination and cross-boundary continuity of care difficult to achieve [25]. Engaging primary care has been an especially difficult task. As primary care providers are independent contractors to the NHS, commissioners have described difficulties harnessing primary care towards a vision of whole system change, implementing complex incentive structures to attempt to persuade them [26].

The most recent planned NHS changes, therefore, involve creation of larger Primary Care Networks, and more devolved, place-based commissioning of services [2]. However, the ‘correct’ level of the system for decisions to be taken is, and has been [27], a key debate in health (and wider publicly-funded) systems internationally. For example, a desire for more local decision-making lies in natural tension with a nationally funded system built on values of equality [27, 28]. Questions still remain, for example, over what the role of NHS England should be in policymaking and commissioning [29]. Particularly, as we start to target more preventative action at a larger, relatively healthier population, there is an argument that this might be more effective if done consistently at a larger (national) scale [17]. On the other hand, commissioning anything at a more national level is likely to be complex and may have unintended consequences [30].

#### Commissioning arrangements for the DPP

The DPP is provided by four behaviour change provider organisations procured by NHS England based on published specifications. The service is commissioned across 41 geographically-defined local sites and each provider is able to provide services at any location within England [31]. Local sites select the most suitable provider for their requirements through a mini competition process [31], so each site ends up with a single service provider. NHS England directly holds the contracts with the four DPP providers and measures referral volume as a main metric of site activity. However, each local site also has a site lead (a commissioner/programme manager) responsible for local implementation, patient identification and referral. NHS England provided local sites with an additional financial resource (£30–£60,000) for implementation in the first year of participation [31].

**Fig. 1:**
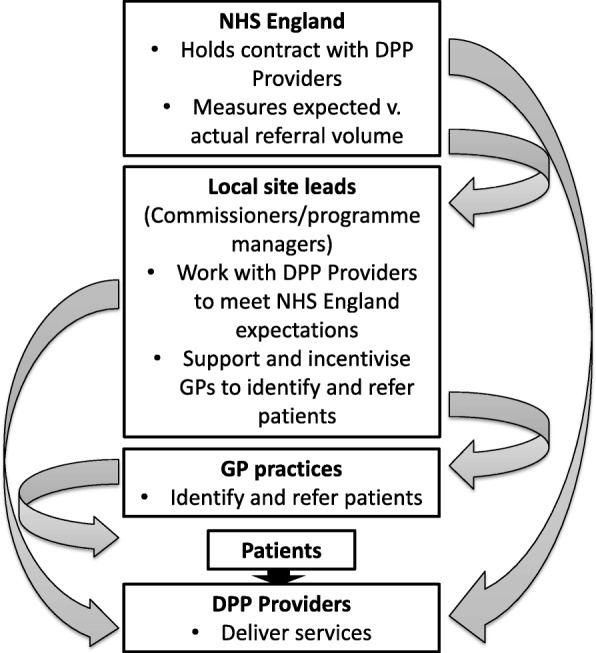
NHS DPP actors

Although guidance was provided by NHS England [32], local implementation was not over-prescribed beyond ensuring the impact of any additional workload on general practices was minimised. The involvement of general practices is limited to screening/identification and referral of patients. Consequently, how local implementation is organised varies from site to site and local site leads have employed a range of incentive and support strategies to help general practices to identify and refer relevant patients to the DPP. Figure 1 illustrates the relationships between the various actors involved in implementing the DPP. As illustrated in Fig. 1, this results in a mixture of hierarchical relationships and direct relationships between NHS England/local site leads and DPP providers.

With these issues in mind, we report on the implementation of the DPP in the NHS in England, and identify lessons learned in addressing the implementation challenges.

## Methods

### Sample and participants

Our intention was to devise a sampling strategy that would provide sufficient case sites for detailed longitudinal exploration by the DIPLOMA evaluation programme as a whole. As such, using the defined geographical areas used for programme provision as the sampling framework we aimed to recruit a mix of case sites varying on characteristics including NHS DPP provider, rural and urban locations, populations with different socio–economic characteristics, as well as those proposing a range of recruitment and incentive strategies. We obtained the prospectus documents prepared by each wave 1 site for providers to review prior to ‘bidding’ for all geographical areas participating in the NHS DPP programme. These were coded and analysed to generate a purposive sample of 16 case sites (*n* = 4 case sites per DPP provider). As the amount of data presented in prospectus documents varied, information on the rural/urban classification of the area and the ethnic composition of the population was obtained from data from the Office of National Statistics 2011 Census [33].

We obtained contact details for the designated lead(s) responsible for local commissioning and implementation at each sampled case site from the NHS DPP National Management team at NHS England. These individuals were contacted by the research team by email and invited to participate in a semi-structured telephone interview. Email invitations included a copy of the participant information sheet. Where more than one lead had been identified at any site, invites were sent to all named leads to maximise data collection opportunities and ensure that individuals involved in all aspects of the implementation of the programme had the opportunity to take part. If no response was received within 3 weeks a follow-up invitation was sent. A consent form was sent in the post or electronically for completion and return prior to the interview taking place.

Interviews aimed to explore the process of local implementation of the NHS DPP including the local organisation of the programme, the expectations of and attitudes to the NHS DPP among local leads, funding, target populations and referral and clinical pathways (see Additional file 1 for topic guide). In total, 20 interviews were conducted covering all 16 of the sampled wave 1 case sites. The majority of respondents (*n* = 17) were senior commissioning managers employed by CCGs. The remaining three interviewees were public health consultants employed by local councils. Interviews were conducted by a member of the research team between November 2017 and January 2018 and lasted between 31 and 66 min dependent on participant availability and their role in relation to implementation.

The interviews were digitally recorded and transcribed verbatim by a transcription service. All transcripts were anonymised and an inductive thematic analysis was undertaken with the aid of NVivo 10 [34]. Using aspects of the constant comparative method of analysis [35], data were coded and explored to identify similarities and differences across interviews, which in turn led to the development of over-arching themes.

During extraction of data from the prospectus and discussions with interview participants it became clear that there were large variations (within, by more local area, and between sites) in how incentives had been implemented and the monetary amount associated with them. Since patient recruitment by general practice is likely crucial to functioning and effectiveness of the programme, we wanted to better understand this variation. We subsequently created a questionnaire to obtain quantifiable incentive data aimed to address inconsistencies (often what was detailed in the prospectus was not what was subsequently implemented) and to explore variations further.

### Incentives survey

In May and June 2018, we contacted all designated local commissioning leads for all the wave 1 and wave 2 defined geographical areas participating in the NHS DPP programme. Each local lead was contacted by email and asked if they could provide more information about the implementation of local incentives. We attached a short questionnaire (see Additional file 1) to be completed and returned by email, or offered to arrange a time to contact and complete over the phone if they preferred. Where additional local lead names were provided these were followed up and contacted. Where multiple responses were received for a site, these were aggregated into a single response; with any clarifications sought from respondents via email. Reminder emails were sent at two and 4 weeks. Any questionnaires not returned by 30th June 2018 were deemed to be non-responses.

## Results

We identified five over-arching implementation themes from our interviews: 1) managing new providers; 2) promoting awareness of services; 3) recruiting patients; 4) incentive payments; 5) mechanisms for sharing learning (which was also identified as an opportunity for addressing the other challenges). Across all of these themes, we found recurring issues of communication, managing relationships, and defining responsibilities.

### Managing new providers

Respondents highlighted a lack of defined responsibility with regards to provider assurance and performance management, and a confused contractual relationship – NHS England as the provider contract holder, but the local site leads expected to manage the contract delivery day-to-day. This appeared to translate into relationship tensions. This left some local leads frustrated at their lack of levers to manage provider performance.

“*At the moment, I feel like we sort of get the worst of both worlds really. We’re asked about assurance and performance management from NHS England. And then if we have a problem with the provider around that, we sort of push back to NHS England and say, can you manage this through the contract? They will then push back to us and say, you manage it locally. You’re having that local arrangement with the provider. And we do have that so … swings and roundabouts, we do have a local arrangement with the provider, so we do understand them probably better on the ground than they do. But you do … lose some of that clout, so I just think it needs to be clear. I wouldn’t say one way is the right way necessarily, but I think the arrangement and the working arrangement could be much better set out.*” (WP22–06, W1)

However, relationships with providers developed over time. For example, local leads expressed some sympathy with the fluctuating level of activity (i.e. numbers of referrals from primary care) that providers had to deal with, partially caused by expectations and incentives placed on site leads and general practitioners (GPs) to refer (discussed in more detail below).

“*We’ve had some teething problems with them [Provider] certainly, and at the moment, they are really struggling to cope with the level of activity coming through. But I think we are potentially responsible for some of that as well … 1, we’ve done such a good job, but 2, we did such a bad job to start with, if you get me, as well … just really setting us all around a steady state and saying, that’s our expectation. We think the provider ought to be doing a bit more to engage with general practice. Then having a bit of a kick up the backside from NHS England saying, no, you need to generate these referrals. Then having generated them, they’ve come through at a volume whereby, we didn’t expect … And that’s left the provider in a tricky position...*” (WP22–14, W2)

Some working relationships between local site leaders and providers were more positive, apparently based on providers’ early actions to engage directly and also to understand the local area, population and needs.

“*They’ve [Provider] been very positive, and they're very nice, and they've employed a coordinator to work more directly with our coordinator. So, yeah, the relationships are good, and we're starting to get referrals through, and they've been fine. I think it's just that, I guess, their application was written assuming that they were competing with three other providers. So it all sounded like it would be no trouble whatsoever. And I think, you know, obviously, reality is a little bit different. But it's been great.*” (WP22–03, W2)

“*They’ve [Provider] been absolutely fantastic, to be honest … they’ve definitely worked with me to look at the site … we had them come into our office and look at all the options off site, why we were looking at the sites in the way we were looking at. Ours is a really huge borough, and so we needed to make sure that there was equity across the whole patch. But also where there’s possible siting it’s better to use the premises that are already there.*” (WP22–11, W2)

### Promoting awareness of services

Awareness of the programme was regarded by site leads as vital for uptake. However, many of the leads indicated that there was a general lack of awareness of the NHS DPP among patients and professionals, including GPs who were responsible for recruiting patients.

“*I think a lot of them [GPs] still don’t know what the programme is … I think they know it’s a lifestyle programme but that’s probably about it*” (WP22–08, W1)

Many sites introduced financial incentives to stimulate this engagement (discussed in detail below). Some sites also worked to provide educational opportunities at a local level to address this.

“*One factor we were concerned about was the actual primary care buy in. We wanted them to be engaged, and understand the benefits of the service, and to make sure that they referred the appropriate patients onto it … We’ve done a lot of clinical education, in terms of we’ve attended protected learning times for GPs and nurses, to make them aware of the service.*” (WP22–15, W2)

With NHS England taking the lead responsibility for the legal contracts with providers, there was initial confusion about who was actually responsible for promotion of the programme: the site lead or the provider? Providers were not resourced to promote their service, but local sites were not initially aware of this.

“*We found it difficult to understand why the provider didn’t, or didn’t seem keen to, actively promote their service … if they don’t advertise, people don’t know they’re there … But then it was made clear to us that actually we had some responsibility for that as well, and actually they, from the provider perspective, they felt that there was little resource in their funding to support promotion of the service.*” (WP22–08, W1)

Many local site leaders felt there was the need for NHS England to implement a national campaign to raise awareness, further increase ‘*brand recognition*’, and aid the engagement of patients (to directly stimulate patient demand).

“ *… nobody knows what it is. If you say to someone send them a text it’s time for the flu jab, they go, yeah, I know what that means. We all see the posters, we’ve seen the national advertising, it’s everywhere. You send a text saying to someone we’ve got a diabetes prevention programme, they have no brand recognition … So there’s something about getting the collateral right in terms of comms*.” (WP22–07, W1)

### Recruiting patients

NHS England’s focus was on encouraging sufficient referral volume when rolling out the service. However, some site leads recognised that quantity of referrals does not necessarily relate to ‘quality’ of referrals. They were concerned that if patients were not motivated this would impact upon their engagement and retention within the programme. This nuance was thought to require a specific, more resource-intensive ‘warm’ recruitment approach to capture the ‘right’ patients.

“*We had decided to actually put some investment into the locally commissioned service in order to make sure that the referrals were good referrals. What we had asked our practices to do is actually call in the patients into the practice, have a brief intervention so that patient is actually motivated to enter into the nine month programme. There’s no point in just sending people if they’re not motivated, if they’re not ready …* ” (WP22–11, W2)

Others opted for a ‘cold’ recruitment approach where patients were identified as suitable via general practice lists and were sent letters in the post to meet referral targets. As well as resources, the method of recruitment chosen was likely to affect flow of patients to providers over time – for example, sending out multiple mail-shots at once could lead to a sudden burst of activity being generated. There was additionally a concern regarding the ability to sustain the number of referrals after initial roll-out.

“*Because they [NHS England] put on pressure to us, there’s not...the only tap that you have is the mail shots, anything else is a slow build up. If you want to turn on the tap you do the mail shots, you’re going to get a response, I don't think it’s ideal but there you go. We have now got a volume of patients coming through.*” (WP22–07, W1)

“*Once practices have done all of their mail-shots, then I think the activity will probably slow down and you’ll hit a steady state which will be obviously much lower than some of the volumes and levels of activity which are coming through at the moment.*” (WP22–06, W1)

Different recruitment approaches were tried out over time, and advice was sought from GPs on occasion. Some sites indicated that a mixed recruitment approach may be necessary in order to ensure the sustainability of referral numbers and to reach out to individuals who may not respond to one or other approach in the hope of addressing equity issues.

“*We take their advice really [GPs] on how they think, a) is the best way to reach them. You know, is it coming into practice to do something? Is it … Initially when we were incentivising them, you know, is it sending a letter? And some practices were, absolutely not. The patients, you know, English is not their first language so they won’t respond to it. They call … telephoned all their patients.*” (WP22–08, W1)

“*I suppose even though it’s [mail shots] worked well in as much as we’ve got the number of referrals that...our target, we’re reaching those targets very well...it’s only 20-25 per cent of the people who have received a letter who are taking up the programme...I suppose we’ve only really hit the motivated people at the moment. So after we’ve done...we’re only going to do this with the GP practices once and then we’re going on to the opportunistic. So we’ve really I suppose hit those people that are motivated and the easy ones really. It will be more difficult to get the others.*” (WP22–14, W2)

### Incentive payments

Early interviews indicated that the money allocated for the implementation of the NHS DPP had been utilised in differing ways to support GP practices to recruit patients. As most areas contain multiple CCGs, interviews had also indicated the possibility of within site variation in the provision of local incentives. Our subsequent survey results helped clarify how these differed. A total 57 individual responses were obtained from 30 sites (out of a total of 41 sites).

NHS DPP funding for implementation was frequently complemented by additional local CCG funding. It was used by the majority of sites as a form of financial incentive for general practices to encourage recruitment. A number of sites also invested in additional resources on top of the recruitment payments, for instance, funding dedicated support for case finding in general practices, administrative and IT support, and postage.

Twenty-seven of the thirty sites offered financial incentives to GP practices. Of the three sites not offering financial incentives, one indicated that this decision was based on feedback from earlier implementing sites which suggested that referral generation had not greatly increased with piloted incentives. For the sites that did offer incentives to GP practices, these incentives were organised in three overarching ways. In some cases there was within-site (CCG level) variation, both of the amount paid, and activity which was incentivised. In general, the amount paid per incentivised activity increased with specificity of the payment type:

1. Seven sites implemented the broadest payment type, similar to a capitation payment, where the GP practice was paid a fixed sum based on the size of their registered patient population identified as eligible for the DPP intervention. Amounts ranged from £0.05 to £0.30 per eligible patient. One CCG offered a £2.05 per patient payment but additional specific criteria needed to be met in order for practices to receive this. Practices had to hold a pre-diabetic register, identify eligible patients by running a search using specified criteria, offer suitable patients referral to the programme, and record outcome using appropriate Read codes [36].
2. Thirteen sites implemented a payment more similar to a fee-for-service payment, where the GP practice was paid per referral letter sent. £1.50 per patient referral invite letter sent was the amount most commonly offered (range: £0.70 to £2.91). One site offered an enhanced referral letter payment of £4.00 which included a follow up phone call and or text message to the patient. A similar enhanced scheme was utilised by one CCG which sanctioned a payment of £5.95 for every patient who was identified as at risk and then contacted and informed about the NHS DPP programme.
3. Nine sites implemented the most specific payment type, similar to a pay-for-performance payment, where the GP practice was paid based on the number of patients they actually referred to the programme. Payments ranged from £2.52 to £45.00 per patient depending on what was locally defined as the actual referral point. One site indicated that in Year 1 they paid £10.00 per actual referral to ‘kick-start’ the programme but had reduced this to £2.00 per referral in Year 2 which was thought to be a more sustainable payment model. Another site offered a two-stage payment, one for referral and then a subsequent payment for those that converted to an initial assessment. They said a few eyebrows had been raised in terms of the amount offered but declined to say how much. They were keen to stress that they have not invested in other support mechanisms so overall costs may be similar to other sites.

Four sites offered other variations on incentive payment. For example, payments were bundled into existing local incentive contracts or as a one-off payment to practices.

Table 1 summarises the variation in incentives offered, by site.

**Table 1:**
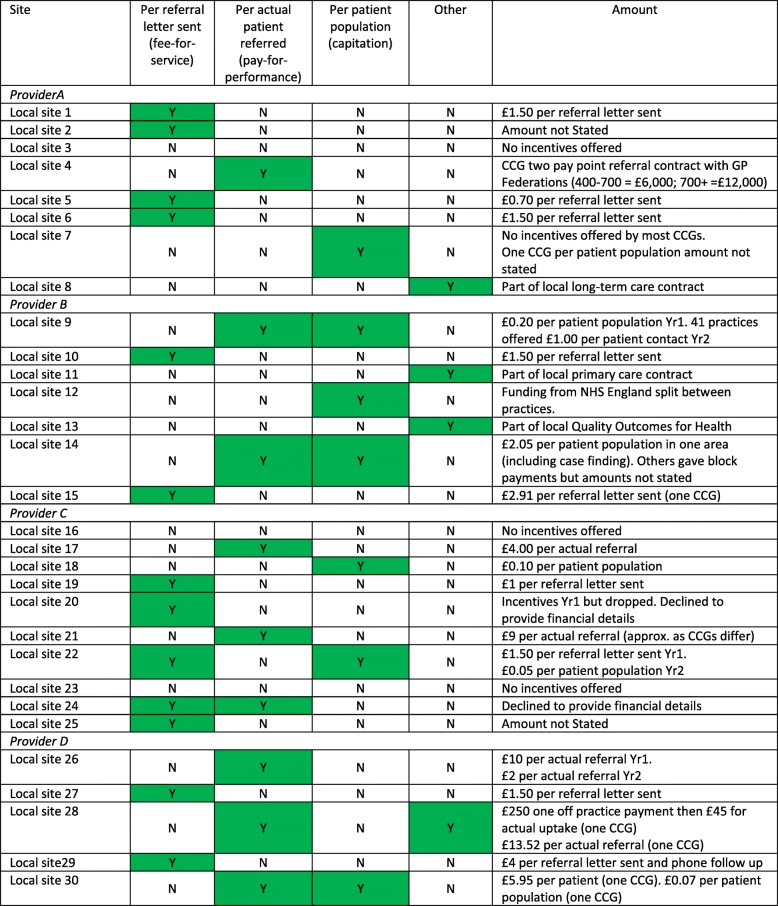
Forms of incentive schemes offered for practices participating in NDPP (by responding site)

### Mechanisms for sharing learning

The majority of leads recognised the importance of sharing knowledge, but the forum was not always available to do so. Within a large area, sharing across sub-sites was deemed important.

“*we made contact with [sub-area – CCG] and tried to get their knowledge and experience from having already done it; but there’s no regular forum where we all get together or where we all feedback on the overall programme; bearing in mind we’ve all got the same provider. Some of that learning and information is quite useful, and maybe it would be a lot more powerful voice if it’s coming, especially in regards to feeding back to NHS England collectively across our STP [Sustainability and Transformation Partnership] footprint area, we’re all saying the same thing.*” (WP22–12, W2)

Learning from other areas was also seen as important, particularly later implementers learning from earlier ones. Again, however, many reported that these learning opportunities were not being made readily available.

“*We try to work with the provider to see if we can find a way for coding to be encrypted on any information they send out … We haven’t found a way to do that but if somebody can find a way please let us know. And I’m wondering if a lot of these things will come out during the process given that everybody’s potentially facing some of these challenges and I guess there might be some sites that work out how to overcome these. That it would be great if the programme, if we could just share all this knowledge amongst all the different sites that are running.*” (WP22–01, W2)

Where shared learning had taken place, the opportunities were regarded as highly beneficial.

“*I think I struggled initially knowing who to speak to. I went to all the NDP [NHS DPP] workshops and regional events and heard a lot about what had happened in phase one and the learning there so that was really useful because I’d sort of got a heads up of what might be some of the challenges so that was useful learning.*” (WP22–05, W2)

Site leads reflected upon some of the challenges that they had experienced and in doing so identified a number of possible opportunities for learning (see Table 2). These included the following possible avenues to support learning: national learning sets; workshops; webinars; regional newsletters; keeping a local ‘lessons learnt log’.

**Table 2:** Potential learning opportunities

Challenge	Opportunity
Uncertainties about optimal and effective content and length of patient documentation e.g. referral and reminder letters	Share documents across sites, and learning from other national initiatives to develop a standardised enhanced format
Many general practice systems are not set up to effectively manage NHS DPP programme	Share standardised coding generated for general practice systems across sites
Challenges working within different governance systems e.g. between CCG/STP/Local Authority/General Practice in the setting up and delivery of the programme	Discussions within and between sites with key stakeholders to develop and implement effective models of multidisciplinary working
Knowing who to speak to e.g. NHS England contact, particularly when individuals change frequently	Ensure frequent communication from NHS England regarding changes
Engaging different populations	Share information between sites regarding implementation of methods to ensure equity of referrals
Overcoming issues related to commissioning model (e.g. getting info from provider)	Clear guidance from NHS England regarding roles and responsibilities
How to support Primary Care	Discussing with other sites the approaches that have been implemented to support Primary Care, including how best to use resources
Recruitment approaches and managing/sustaining trajectory of referrals	Share learning across sites as to what approaches worked best. Identifying benefits/sustainability of ‘warm’ vs ‘cold’ approaches

## Discussion

### Summary of findings

The NHS in England implemented a national DPP programme. We conducted interviews with site leads to explore the challenges around the implementation of this programme, with the aim of providing lessons for future practice. We identified five over-arching areas of learning for implementing a large-scale programme like the DPP: managing new providers; promoting awareness of services; recruiting patients; incentive payments; mechanisms for sharing learning (which was also identified as an opportunity for addressing the other challenges). In general, tensions between hierarchical actors appeared to be caused by a lack of clear roles/responsibilities between hierarchical actors, and lack of communication. Both local sites and the national NHS coordination team gained experience through learning by doing, and some initial tensions with roles and expectations have been worked out ‘live’. However, future implementers might wish to ensure responsibilities for each actor are more clearly defined prior to implementation. They may also be advised to ensure early engagement with new providers, offer mechanisms/forums for sharing learning, offer advice on incentive payments (or randomly assign to robustly test effects [37]), and ensure public and professional awareness is prioritised.

### Strengths and weaknesses

We combine two methodologies to offer implementation insights. Our qualitative interviews drew on a strong sampling methodology to ensure a mix of perspectives and insights were reflected. This was complemented by a survey designed to systematically unpick the variation in incentive payments offered to GPs for referring patients that became clear during interviews. We obtained responses to this survey from over 75% of sites operating in England. However, as we did not obtain complete coverage and we undertook selective sampling for qualitative work, there is a risk that our findings may not be generalisable.

This paper provides only a snapshot of the implementation issues faced by sites at a single point in time, and issues may change over time. In August 2018, for instance, NHS England announced the introduction of a new service specification to enable the NHS DPP to offer greater uptake and access for service users. The aim of the new framework is to improve take-up and adherence, including better targeting of working age populations and addressing delays associated with running courses in rural areas. The most notable change from the previous framework is the inclusion of the offer of a remote/digital service as an adjunct to face-to-face delivery. The new framework will be operational from August 2019 and we intend to explore the process of its implementation across our selected case sites in future work. In doing so, we will explore the implementation of the new framework and its associated delivery arrangements, exploring how local organisation of the programme has changed (if at all), and with what consequences (anticipated or unintended).

### Comparison to existing literature

Much of the international literature identifying the key factors that lead to successful implementation of diabetes prevention programmes has focused on intervention delivery and fidelity [13, 38]. Exceptions are two articles detailing researchers’ experiences of implementing the original Finnish DPP in Australia [39, 40]. The authors outline potential success factors, including: demonstrating relative advantage over the status quo; compatibility with health professionals’ values and behaviours; lack of intervention complexity; trialability and observable results; and a conducive atmosphere for policy change [39]. In practice, adherence to the original intervention guidelines (for intervention effectiveness beyond the controlled clinical trial to more ‘real world’ settings) and co-operation between policymakers, implementers and researchers over a number of years were deemed enablers of these factors [40]. These studies might highlight further implementation challenges for the NHS DPP with a current lack of effectiveness evidence in this specific context (assuming that the proven effectiveness in other contexts is not known or accepted by the GPs recruiting to the programme).

There has been less focus on the strategies and processes by which target populations are identified and then referred into the programme, and how hierarchical actors interact. Indeed, a systematic review looking at critical success factors for implementing programmes in real-world settings found a lack of information on the nature and type of recruitment strategies deployed [13]. We add to this literature by looking at scale-up and national roll-out at an organisational level. We offer prescriptive learning for those implementing a national prevention programme, particularly relevant if outside providers are used to provide extra capacity.

A recent study examining implementation of the NHS England new care models ‘Vanguard’ programme also focused on practical learning from implementation of a large programme at an organisational level [41]. The Vanguard programme focussed on improving integration across existing local health and social care providers. The authors similarly offered advice on communication and clear decision-making roles as key learning areas, so it is questionable how much of the challenges we describe are a general implementation issue for NHS England and how much specific to diabetes or prevention. Where the DPP programme differs from the Vanguards, however, is its involvement of brand new provider organisations, and the increased involvement of national commissioners. Arguably, this will be required more and more as the health system expands its horizon to focus on currently healthy individuals and preventing potential future disease. We provide insights directly relevant to interacting and contracting with providers of this type.

The extent to which centralised and decentralised health systems differ on outcomes is an on-going debate. Despite the recent political move towards devolution in many areas, a report by the Health Foundation concluded that there is an overall lack of empirical evidence that decentralised systems outperform centralised ones [42]. We add to this literature providing direct examples of how tensions between local and national decision-making and implementation play out in practice. The perspectives of the site leads we interviewed provide suggestions on how these issues could potentially be lessened.

### Policy implications

Certain specific actions such as a singular branded advertising campaign are likely to be more effective when taken at a national rather than local level. NHS England has implemented a national advertising programme to promote the NHS DPP following feedback from our research team of the concerns of the sites around programme awareness. However, additional complexities must be taken into account. In this instance NHS England had initially made the decision not to advertise the programme nationally due to the phased roll-out design of the DPP. A national advertising campaign run at the start of the programme would have likely stimulated demand in areas where the DPP was not yet available. This highlights the importance of considering both the intended and unintended consequences of various methods of programme design.

The ‘right’ level of decision-making for other aspects of programme decisions is much less clear. For example, it is not immediately clear whether it is preferable to hold provider contracts nationally or locally as both national oversight and standards of provision as well as local insights into issues such as specific population needs are valued. Site leads were similarly unclear on how this contracting would work best, but suggested they would at least value clarity of the expectation of their specific roles from the outset. In practice, it is unlikely that any new programme will perfectly predict the practical issues that will be faced during implementation. Implementation is not linear but dynamic and as such local-level adaptation can be important for maximising effects, encouraging uptake and sustainability [43]. Having a mechanism in place so that actors can quickly adapt to the challenges they face should be a priority for responsive roll-out.

The majority of sites opted to employ financial incentives to engage and resource primary care providers tasked with referring patients into the programme. We are unable to determine the relative effectiveness of these variable incentive payments. However, the issue they all aim to address, i.e. obtaining widespread ‘buy-in’ to a new initiative from a fragmented, independent-contractor primary care system, is one we have identified previously [44]. The plan to harness primary care through mandated Primary Care Network (a mechanism for contracting multiple primary care providers at once) involvement proposed in the NHS Long Term Plan might be one way to address this issue [2].

### Future research

The role and value of financial incentives for stimulating wanted behaviour down through hierarchical levels of actors is something we plan to explore in future work. In the DPP we have identified variation in incentive payments across geographical area and over time which we plan to exploit to identify effectiveness on desired outcomes, for example in relation to potential variations in recruitment rates as roll out unfolds.

## Conclusions

Implementing a national disease prevention programme is a major task, and one that will be increasingly faced by health systems globally as they aim to adjust to demand pressures. We provide practical learning opportunities for the wider uptake and sustainability of programmes like the NHS DPP. Future implementers might wish to ensure responsibilities for each actor are more clearly defined prior to implementation, ensure early engagement with new providers, offer mechanisms/forums for sharing learning, provide advice on incentive payments (or randomly assign to robustly test effects), and ensure public and professional awareness of the programme is prioritised.

## Supplementary information

**Additional file 1.** Interview topic guide.

## Data Availability

All available data can be obtained from the corresponding author. All data will be shared in a way that safeguards the confidentiality and anonymity of respondents.

## Abbreviations

CCG: Clinical Commissioning Group
DIPLOMA: Diabetes Prevention – Long term Multimethod Assessment
DPP: Diabetes Prevention Programme
GP: General practice
GPs: General Practitioners
NHS: National Health Service
STP: Sustainability and Transformation Partnership
WHO: World Health Organization
