# Supplementary material for: Implementing a national diabetes prevention programme in England: lessons learned
Source: BMC Health Serv Res. 2019 Dec 23;19:991. doi: 10.1186/s12913-019-4809-3 (PMC6929377; doi:10.1186/s12913-019-4809-3)
Supplement: Supplementary file 1 — Additional file 1. Interview topic guide. [file 12913_2019_4809_MOESM1_ESM.docx]

# Interview topic guide

**What are the barriers and facilitators to the implementation of the**

**NHS DPP within areas**

Topic Guide

**Introduction**

- Explanation of ethics, consent and confidentiality of interview and analysis.
- Explanation of objective of acceptability study
- Structure and duration of the interview.
- Any questions?

**What was your role in relation to establishing the local NHS DPP service?**

- How was the local provider selected
- What challenges were faced?
- What went well?
- How does NHS DPP fit with existing local service provision for diabetes prevention and weight management?

**What are your expectations for the NHS DPP locally?**

- Target populations
- Perceived benefits
- Perceived risks

**What are your views on the implementation of the NHS DPP into routine general practice?**

- How do you think NHS DPP has impacted ways of working in general practice?
- How are referral and clinical pathways being embedded?
- Are resources available to support general practices
- Do you have any concerns?

**What are your views on the long term sustainability of the NHS DPP?**

- Any concerns?
- Can you identify any barriers to sustainability?
- Any suggestions about future development of the local NHS DPP service?

**What advice would you have for other areas wishing to implement NHS DPP locally?**

**Is there anything else you want to ask or say?**

# Incentive questionnaire

National Diabetes Prevention Programme Evaluation

**Incentives Questionnaire**

1. Have any incentives been made available to support general practices generate referrals? Yes/No

• **If yes** what have these entailed?

____________________________________________________________________

____________________________________________________________________

- **If yes** how have they been funded?

____________________________________________________________________

____________________________________________________________________

• **If no** is there a reason that you have not offered any incentives?

____________________________________________________________________

____________________________________________________________________

2. Have any other resources been made available to support general practices generate referrals? Yes/No

• **If yes** what have these entailed?

____________________________________________________________________

____________________________________________________________________

• **If yes** how have they been funded?

____________________________________________________________________

____________________________________________________________________

- **If no** is there a reason that you have not offered any other resources?

____________________________________________________________________

____________________________________________________________________

3 Have all CCGs in your area provided similar support to general practices? Yes/No (please detail)

____________________________________________________________________

____________________________________________________________________
